# Counterfactuals of effects of vaccination and public health measures on COVID-19 cases in Canada: What could have happened?

**DOI:** 10.14745/ccdr.v48i78a01

**Published:** 2022-07-07

**Authors:** Nicholas H Ogden, Patricia Turgeon, Aamir Fazil, Julia Clark, Vanessa Gabriele-Rivet, Theresa Tam, Victoria Ng

**Affiliations:** 1 Public Health Risk Sciences Division, National Microbiology Laboratory, Public Health Agency of Canada, St-Hyacinthe, QC and Guelph, ON; 2 Office of the Chief Public Health Officer, Public Health Agency of Canada, Ottawa, ON

**Keywords:** COVID-19, Canada, vaccination, public health measures, counterfactual, modelling

## Abstract

This study illustrates what may have happened, in terms of coronavirus disease 2019 (COVID-19) infections, hospitalizations and deaths in Canada, had public health measures not been used to control the COVID-19 epidemic, and had restrictions been lifted with low levels of vaccination, or no vaccination, of the Canadian population. The timeline of the epidemic in Canada, and the public health interventions used to control the epidemic, are reviewed. Comparisons against outcomes in other countries and counterfactual modelling illustrate the relative success of control of the epidemic in Canada. Together, these observations show that without the use of restrictive measures and without high levels of vaccination, Canada could have experienced substantially higher numbers of infections and hospitalizations and almost a million deaths.

## Introduction

The severe acute respiratory syndrome coronavirus 2 (SARS-CoV-2) has caused a pandemic because 1) it is highly transmissible from human to human and 2) at the time of the spillover to humans, there was no known immunity to the virus in the global human population. Pandemics end only when there is a sufficient proportion of the population immune (following infection and/or vaccination) to drive the causal pathogen to extinction or to some form of global endemic state that arises due to waning immunity in the human population and/or emergence of immune escape variants. The ”wild type” (WT) variant that emerged in late 2019 had a basic reproduction number (*R_0_*) of approximately two in high-income countries (i.e. on average, every infected person will infect two people in a population with no immunity and with no public health [PH] measures in place). With an *R_0_* of approximately two, and without vaccines, more than 50% of the population needs to acquire infection and become immune before the pandemic begins to come under control, and approximately 75% of the population has acquired the infection by the time the pandemic ends ((1)). Due to the relatively high virulence of SARS-CoV-2—an infection fatality rate approaching 1% and an infection-hospitalization rate approaching 10% (see public health measures section) and a lack of effective therapies and vaccines—the consequences for Canadians, and the Canadian health system, of unrestrained SARS-CoV-2 spread in 2020 were dire (**Table 1**) ((1)). Such a situation and resultant consequences were seen in Italy in early 2020 ((2)). In this article, the coronavirus disease 2019 (COVID-19) epidemic that occurred in Canada, impacted by public health measures and vaccination, is described and compared with outcomes in similar countries (the first section of the study), and then compared with possible alternative outcomes in Canada using modelling of counterfactual scenarios for different levels of vaccination and PH measures than those actually implemented (the second section of the study).

**Table 1:** Counterfactual total numbers of expected cases, hospitalizations and deaths from coronavirus disease 2019 from modelling compared to observed numbers

Outcome	Counterfactual without public health measures or vaccines	Observed as of April 24, 2022, with public health measures and vaccines
Cases	Up to 34 million^a^	3.3 million^a^
Hospitalizations	Up to 2 million	150,602
Deaths	Up to 800,000	38,783

^a^ Reported cases mostly do not include approximately one-third infections that would be asymptomatic, which would mostly go undetected by surveillance. Many mild immunity-breakthrough cases during the Omicron waves are also not captured in surveillance data but are included in counterfactuals

## Description of the evolving epidemic, public health measures and evidence

### Evolving knowledge of the epidemiology of severe acute respiratory syndrome coronavirus 2

Key epidemiological variables for planning and modelling include estimates of the speed of transmission (particularly *R_0_*) and of the severity of infections such as case or infection-hospitalization and fatality rates. Since early March 2020, the Public Health Agency of Canada has conducted daily literature searches to obtain the most up-to-date estimates of these values. Initially, estimates of *R_0_* (approximately 2–3) and case-hospitalization and fatality rates (10% and 1.2%, respectively) were obtained from studies in China ((3)). Given that transmission varies depending on the rate of contact between people ((4)), *R_0_* values vary depending on the country or region in which they are measured ((5)). Overall fatality rates are also dependent on the demography of the country studied, due to age-varying fatality rates ((6)). The estimated values of the key epidemiological variables varied over time. For example, it became evident that up to 30% of infections are asymptomatic and unlikely to be efficiently detected in surveillance systems ((7)). Furthermore, variants emerged that were increasingly transmissible (WT < Alpha < Delta < Omicron: *R_0_* increased from 2–3, to 3.5, to 5–7, and then to approximately 10) ((8)). Except for Omicron ((9)), these variants were also more virulent than the original WT strains ((10,11)).

### Public health measures to control coronavirus disease 2019

Canadian pandemic planning that focused on a pandemic influenza virus as the most likely cause–response to its emergence would involve treatment of severely affected people with antivirals until the vaccine industry develops a modified influenza vaccine to control infection, as occurred during the H1N1 pandemic ((12)). In March 2020, Canada was faced with a highly transmissible and virulent pathogen (infection fatality rate [IFR] of approximately 1% compared to 0.04% for seasonal influenza) for which there was no natural immunity, no vaccine (or immediate prospect of a vaccine) and no effective antivirals. Therefore, in March 2020 and until vaccines were developed, the only available interventions were non-pharmaceutical interventions (NPIs or PH measures) that prevent transmission in the population, either by 1) reducing the frequency of contacts between infected and uninfected people, or 2) reducing the probability that transmission occurs when infected people come into contact (directly or indirectly) with uninfected people. The “frequency of contact-reducing” measures are those that target people known to be, or most likely to be, infected (testing to detect and then isolate cases, and contact tracing and quarantine of contacts) ((13)), and restrictive closures that aim to reduce contacts more widely in the population, which included closures of schools, ”non-essential” businesses and leisure/recreation venues, teleworking, limitations on religious and private gatherings and curfews, etc. ((14)). The “transmission probability-reducing” measures are those personal measures such as distancing, hand-washing, screens and masks that limit spread of droplets ((14,15)) and enhancements to ventilation that reduce the density of aerosol-borne virions ((16)). In addition, international and domestic travel restrictions were used to limit introduction of infection into locations (e.g. the Canadian Territories and Atlantic provinces) to where it had not yet spread or was at low prevalence and slow the rate of introduction of infection to the population more generally. In this article, the use of these NPIs is tracked over time using a stringency index, which is a semi-quantitative combination of information from nine different PH interventions (school closure, workplace closure, cancelling public events, restrictions on gathering sizes, closure of public transport, stay at home requirements, restrictions on internal movement, restrictions on international travel and public information campaigns) obtained from the Government Response Tracker ((17)).

### Medical counter measures—therapeutics and vaccines

According to the Pan-American Health Organization review on COVID-19 therapeutic options, hundreds of therapeutic options are being assessed through more than 10,000 studies ((18)). Among them, six have been approved to date in Canada ((19)). These include monoclonal antibodies that aim to prevent SARS-CoV-2 virus from infecting healthy cells. In Canada, four anti-SARS-CoV-2 spike protein monoclonal antibody therapies have been approved. Three monoclonal antibody therapies have been approved for treatment in people with a higher risk of being hospitalized or dying due to COVID-19, because of their age or medical conditions: casirivimab/imdevimab; bamlanivimab; and sotrovimab. In addition, cilgavima/tixagevimab (Evusheld^TM^) is approved for the prevention of COVID-19 for people with weak immune systems, or for those whom vaccination is not recommended. Some of these drugs might lose efficacy against the Omicron variant (or particular sub-lineages) due to multiple mutations in the spike protein ((20,21)). Two antiviral drugs, nirmatrelvir/ritonavir (Paxlovid^TM^) and remdesivir (Veklury^®^), which prevent virus replication, have been approved in Canada. Utilization of these antivirals is limited due to a combination of issues regarding efficacy, interactions with other pharmaceuticals and limitations on which and when COVID-19 patients should receive them. The development of vaccines has been a far greater success story; the mRNA vaccines have been highly effective against both infection and severe outcomes for WT, Alpha and Delta variants ((22–24)). Waning of immunity against infection became evident over a period of a few months following vaccination (although less so in Canada where most received an initial two doses at an extended three-month interval) ((25,26)). Some waning of immunity against severe outcomes is also thought to be occurring, but this appears to be very slow and to occur to a lesser extent, and a third vaccine dose provides higher and more sustained protection ((9,24,26,27)). The emergence of the Omicron variant changed the landscape of the role of vaccines as a means of controlling the epidemic because of its capacity to significantly escape vaccine-induced immunity to infection, with vaccine effectiveness of two doses against infections falling from approximately 90% for the Delta variant of concern (VOC) to 30% or less for Omicron ((24,26)). Vaccines continue to protect against severe outcomes from infections with all variants, including Omicron, particularly after a third dose ((24,26)).

### Chronology of the epidemic and public health measures in Canada

**Figure 1:**
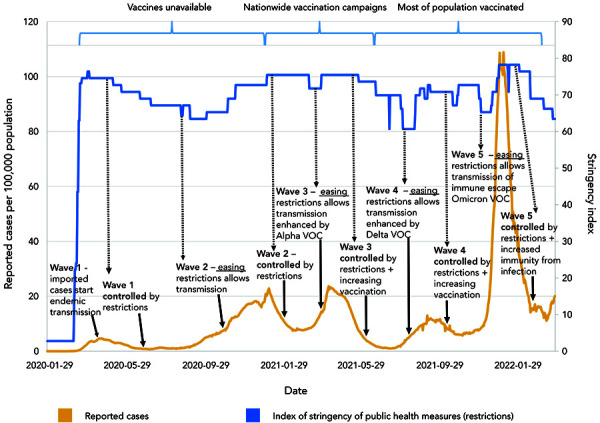
Chronology of the coronavirus disease 2019 epidemic, and public health responses, in Canada up to April 1, 2022^a^ Abbreviation: VOC, variant of concern ^a^ The timeline is curtailed due to reductions in national surveillance

**Figure 2:**
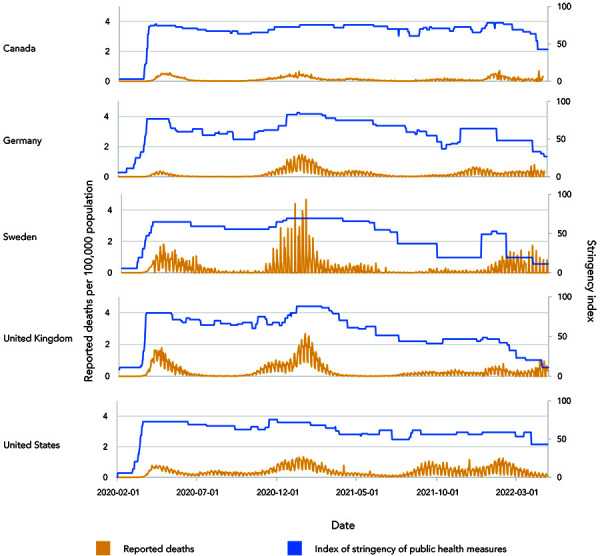
Comparison of the daily reported deaths per 100,000 population and stringency of public health measures in Canada and other high-income countries^a^ ^a^ Surveillance data from ((34))

In the absence of vaccines, two possible control strategies were considered: 1) eradication and prevention of importation, often called the Zero-COVID strategy (see Alternative management of the epidemic section), largely achieved by the Atlantic provinces and Territories for most of the pandemic; or 2) suppression of transmission so that healthcare capacity was not exceeded (the strategy applied in the larger provinces for most of the pandemic). Having observed the severe impact of initially unrestrained SARS-CoV-2 transmission in Italy, when transmission within Canada was recognized and the first wave became evident an initial period of restrictive closures was instigated to pause the epidemic, enhance surveillance and allow alternative NPIs to be resourced and implemented (**Figure 1**). As cases in surveillance began to decline, modelling studies were conducted to estimate the proportions of cases detected and isolated and contacts traced and quarantined that were needed to control transmission if restrictions were to be lifted ((13,28,29)). After the lifting of restrictions in early summer 2020, transmission in the larger provinces began to resurge, indicating that test-and-trace capacity was not sufficient to control the epidemic, and eventually restrictions were reintroduced to safeguard healthcare capacity ((30)) (Figure 1). Throughout the pandemic, this cycle of lifting of restrictions followed by a resurgence of the epidemic followed by reintroduction of restrictions has been a feature of control in the larger provinces (Figure 1). The effect of lifting of restrictions on transmission was exacerbated by the invasion and spread of more transmissible VOCs; Alpha VOC emerging with wave three in spring 2021, and Delta VOC emerging with wave four in late summer/fall 2021. As the vaccines rolled out in 2021, it was hoped that restrictions could be lifted permanently, and many provinces made plans to do this when target percentages of the population were vaccinated. However, the emergence of the more transmissible Alpha and Delta variants meant that higher percentages of the population needed to be vaccinated to allow restrictions to be lifted. Consequently, reintroduction of restrictions was needed to control the waves caused by the Alpha and Delta variants. Most recently, the Omicron variant invaded and spread within Canada in late 2021/early 2022. This variant had characteristics of lower virulence but immune escape. These characteristics were expected from an evolutionary standpoint ((31)); the latter limiting the capacity of the vaccines to control transmission. The combination of high transmissibility and relatively low efficacy of two vaccine doses in preventing the transmission of this variant meant that, despite reduced virulence, healthcare capacity was again challenged and restrictions had to be reintroduced. It is likely that this variant has infected a high proportion of the Canadian population. In a questionnaire study, one-in-five Canadians reported COVID-19 infection in their household since December 1, 2021 ((32)), while in blood donors, seropositivity due to infection rose from 6.4% in December 2021 to 23.7% in mid-February 2022 ((33)). This unprecedented rate of infection during the Omicron wave, combined with the high percentage of the population with two or more vaccine doses (**Table 2**), has brought the immunity of the Canadian population to levels that, at the time of writing, are likely to mean that restrictions can be lifted long-term in Canada (and in many countries across the world), providing that another VOC, that escapes immunity and is virulent, does not emerge. The introduction of vaccines has meant that post-vaccination immunity, rather than simply post-infection immunity, will permit lifting of PH measures, while prior to sufficient levels of immunity being reached, restrictive PH measures have kept the epidemic under control and together this approach has limited severe outcomes and deaths (Table 1). Overall, comparisons of deaths in Canada to those in other high-income countries (**Figure 2**), selected because their levels of public health measures stringency and of vaccine uptake were somewhat different to those in Canada (Table 2), illustrate the relative effectiveness of the Canadian response.

**Table 2:** Cumulative numbers, as of April 20, 2022, of reported deaths due to coronavirus disease 2019 per 100,000 population in countries that did and did not adopt a Zero-COVID approach to managing the pandemic^a^

Country	Cumulative deaths per 100,000 population	Percent of the population vaccinated with two doses
**Did not** adopt a Zero-COVID approach^b^		
Canada	101.3	82%
Denmark	103.7	82%
Germany	159.3	77%
Sweden	183.1	75%
France	214.6	78%
United Kingdom	259.8	73%
Belgium	268.7	79%
United States	291.9	66%
**Did** adopt a Zero-COVID approach		
New Zealand	11.7	80%
Singapore	24.2	90%
Australia	26.7	83%
South Korea	42.2	87%

Abbreviation: COVID, coronavirus disease

^a^ Percentage coverage with two vaccine doses is also shown. Data from ((34))

^b^ As a country as a whole

### Alternative management of the epidemic

Early in the pandemic, it was suggested by some that COVID-19 might be no more serious than seasonal influenza; however, in high-income countries such as Canada, with often relatively older populations, the IFR for COVID-19 in non-immune people has been approximately 1% ((10,11,35)), while for seasonal influenza in the United States, the case-fatality rate is approximately 0.1% ((36)) with an IFR of approximately 0.04% accounting for an estimated 70% of influenza cases that are asymptomatic ((37)). Despite this, some advocates have proposed that management of the pandemic as occurred in Sweden, where management initially relied on voluntary efforts by the public rather than mandatory restrictions, would have been preferable. In fact, Canada has had a low death rate compared with other high-income countries, and a rate approximately a half of that reported in Sweden (Figure 2; Table 2). Counterfactual studies suggest that application of the approach taken in Sweden to countries such as the United Kingdom and Denmark would have resulted in approximately double the number of deaths seen in these countries ((38)). Early in the pandemic, some early, low estimates of COVID-19-specific death rates in North America, particularly for younger age groups, combined with concerns of unintended mental and physical health consequences of restrictive closures, led to the idea of applying restrictions (“shielding”) only to the most vulnerable elderly age groups, allowing younger age groups to live a more normal life ((39)). It became clear, however, that this approach would require shielding to be extended to include much younger age groups (45 years of age and older), which would be impractical and still result in severe outcomes with high mortality rates in all age groups ((40)).

A Zero-COVID strategy was implemented by some countries (e.g. Australia, New Zealand, Singapore) and in the Atlantic Provinces and Territories of Canada, earlier in the pandemic. The objective of the strategy is to completely stop transmission by aggressively using PH measures such as mass testing, contact tracing, border measures and, when necessary, lockdowns, to eliminate new infections and allow a return to normal economic and social activities. Those jurisdictions and countries that adopted this approach were, for the most part, those with limited spread of SARS-CoV-2 when responses began, and with opportunities (e.g. for the island states of Australia and New Zealand) for ease of control of imported cases. As the Omicron variant emerged, most of these countries experienced major outbreaks and have now abandoned this approach; however, this approach allowed vaccination levels in their populations to rise to high levels before significant transmission occurred, therefore limiting the burden on the health system and the numbers of deaths that occurred (Table 2).

## Counterfactual modelling

### Methods

A modelling study is presented to illustrate the importance of both PH measures and vaccination in limiting severe COVID-19 outcomes and deaths in Canada. The study used an agent-based model of a representative 100,000 individuals of the Canadian population ((28,41)). The model was modified to simulate the epidemic in Canada up to the time of writing (April 2022). The model incorporated simulation of the implementation and lifting of the PH measures used (Figure 1), vaccination rollout (first, second and third doses by age groups and priority groups), invasion of the Alpha, Delta and then Omicron BA.1 variants, vaccine effectiveness against infections and severe outcomes specific to each variant, protection against reinfections of the same or a different variant and waning of immunity following vaccination and natural infection. Many parameter values were obtained from the literature, but some were obtained by fitting the model to surveillance and hospitalization data (full details are provided in **Supplemental material**). There were eight scenarios including the baseline (S1), in which an approximation of the actual implementation/lifting of PH measures (including a final complete lifting in March 2022) and vaccination of the population were modelled; and then seven counterfactual scenarios: 1) S2: a worst-case scenario in which no PH measures or vaccinations were implemented; 2) S3: a scenario in which the PH measures were implemented but there were no vaccinations; 3) S4: a scenario in which there were no PH measures but vaccines were administered as observed; and four scenarios in which vaccines were administered as observed and PH measures were also implemented as observed but were lifted early on 4) S5: July 1, 2020 (after the first wave); 5) S6: March 1, 2021 (after the second wave); 6) S7: July 1, 2021 (after the third, combined WT and Alpha variant wave); and 7) S8: November 1, 2021 (after the fourth, Delta variant wave).

### Results

**Figure 3:**
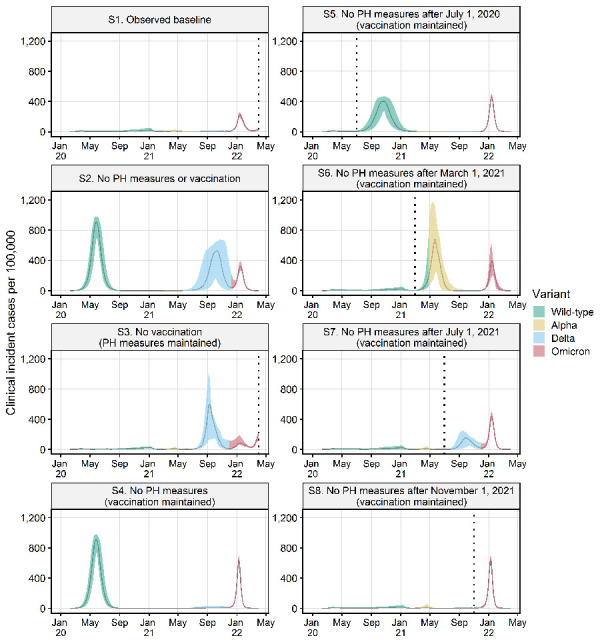
Number of symptomatic infections estimated for seven counterfactual scenarios with different combinations of public health measures and vaccinations to those in the observed baseline scenario^a^ Abbreviation: PH, public health ^a^ Vertical dotted lines indicate the timing of lifting of all public health measures in the baseline, the no-vaccination scenario and four counterfactual scenarios with progressive PH measures lifting. Graphs show the median and 95 percentile values for 100 model runs. The dominant SARS-CoV-2 variant (i.e. more than 50% of cases) for each time period is shown

**Figure 4:**
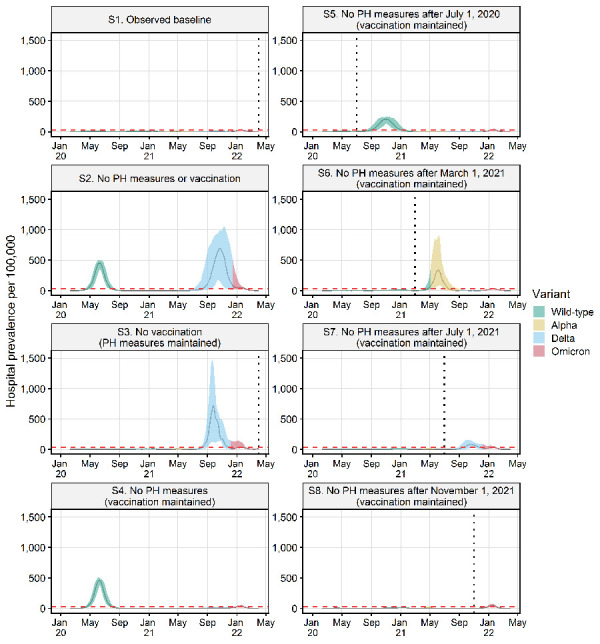
Number of hospitalized cases estimated for seven counterfactual scenarios with different combinations of public health measures and vaccinations to those in the observed baseline scenario^a^ Abbreviation: PH, public health ^a^ Vertical dotted lines indicate the timing of lifting of all public health measures in the baseline, the no-vaccination scenario and four counterfactual scenarios with progressive PH measures lifting. Graphs show the median and 95 percentile values for 100 model runs. The dominant SARS-CoV-2 variant (i.e. more than 50% of cases) for each time period is shown. The red horizontal dashed line shows estimated hospital capacity in Canada

The simulations show that the combination of PH measures and vaccinations that occurred in Canada resulted in far fewer infections, hospitalizations and deaths than in the counterfactual scenarios in which other decisions were made on rollout of vaccines and/or implementation of PH measures (**Figure 3** and **Figure 4**; **Table 3**). In the absence of PH measures and vaccinations (S2), a very large initial wave far exceeded hospital capacity as did a subsequent large Delta-driven wave as immunity waned, and this resulted in a very high number of hospitalizations and deaths (Table 1). In the absence of vaccination, but with PH measures maintained (S3), a very large Delta-driven wave occurred. In the absence of PH measures but with vaccination in place (S4), similar to S2, a very large initial wave in hospitalization would have been observed but the vaccination rollout would have prevented a subsequent Delta-driven wave from occurring. Early lifting of PH measures (S5 to S8) resulted in the resurgence of the epidemic at various points in time corresponding to the timing of lifting, with healthcare capacity being exceeded. The earlier measures were lifted, the worse were the outcomes in terms of hospitalizations and deaths. Lifting after the second wave (S6) coincided with the introduction of a more transmissible and virulent Alpha strain, causing higher hospitalizations and deaths than lifting earlier after the first wave when the WT strain was dominant (S5), whereas lifting after the third wave (S7) caused fewer hospitalizations and deaths despite a more virulent Delta strain in circulation due to higher vaccination coverage. As Omicron is less virulent than all the other strains that have emerged in Canada, a lifting after the fourth wave (S8) would have caused a high number of infections but considerably lower number of hospitalizations compared with the other counterfactual scenarios (Figure 3 and Figure 4). The baseline scenario (S1), modelled on an approximation of actual vaccination and PH measures in Canada, was the only scenario in which hospitalizations were consistently below the hospital bed threshold.

**Table 3:** Key metrics (median and 95 percentiles for 100 model runs) of cases^a^, hospitalizations and deaths estimated by the agent-based model simulations for the observed baseline and seven counterfactual scenarios for the period February 7, 2020 to March 31, 2022

Transmission control methods in the scenarios and outputs of modelling	Counterfactual scenarios							
	S1 Observed baseline	S2 No PH measures or vaccination	S3 No vaccination (PH measures maintained)	S4 No PH measures (vaccination maintained)	S5 No PH measures after July 1, 2020 (vaccination maintained)	S6 No PH measures after March 1, 2021 (vaccination maintained)	S7 No PH measures after July 1, 2021 (vaccination maintained)	S8 No PH measures after November 1, 2021 (vaccination maintained)
Vaccination rollout	Yes	No	No	Yes	Yes	Yes	Yes	Yes
Lifting of PH measures	March 31, 2022	No PH measures	March 31, 2022	No PH measures	July 1, 2020	March 1, 2022	July 1, 2022	November 1, 2022
Clinical cases per 100,000^b^	12,001 (10,028–15,306)	90,154 (89,299–91,277)	38,858 (29,438–43,633)	59,574 (58,509–61,940)	44,746 (43,783–45,556)	47,472 (39,046–52,298)	25,368 (22,115–27,848)	17,983 (16,139–20,842)
Asymptomatic cases per 100,000^b^	47,638 (44,775–51,455)	113,752 (110,854–117,951)	58,754 (52,099–60,876)	108,293 (107,001–111,504)	90,302 (89,493–91,334)	92,660 (74,662–103,826)	84,869 (81,558–87,347)	81,098 (79,752–83,044)
Hospitalizations per 100,000	256 (182–387)	4,715 (4,572–4,918)	2,529 (1,541–3,225)	2,246 (2,136–2,348)	1,619 (1,541–1,722)	1,469 (871–2,150)	601 (500–710)	324 (240–438)
ICU admissions per 100,000	74 (48–111)	1,428 (1,360–1,489)	779 (455–988)	681 (626–724)	498 (452–557)	446 (249–681)	174 (140–212)	93 (66–134)
Deaths per 100,000	48 (32–76)	2,034 (1,938–2,115)	947 (563–1,301)	849 (803–899)	583 (538–634)	350 (182–603)	131 (101–163)	70 (47–92)

Abbreviations: ICU, intensive care unit; PH, public health

^a^ Cases include reinfections and vaccine breakthrough cases, which occurred particularly during the Omicron-driven waves

^b^ Cases are higher than the model population (100,000) in some scenarios due to reinfections in the population

## Discussion

The review and analyses here underline the possibly catastrophic outcomes of the epidemic in Canada, had a combination of non-pharmaceutical PH measures and vaccinations not been implemented to control it. Public health measures, particularly measures that restricted contact between people, maintained control of SARS-CoV-2 transmission until levels of immunity in the population from a combination of high levels of vaccination and infections were sufficient to allow restrictions to be lifted. The relative effectiveness of the response to COVID-19 in Canada is illustrated by the substantially fewer deaths that have occurred in Canada compared with other similar countries. The success of the response is also illustrated by the modelled counterfactual scenarios. While non-pharmaceutical PH measures and the vaccination rollout individually contributed to minimizing severe outcomes, counterfactual modelling suggests that it was the combination of the two that limited morbidity and mortality in the Canadian population. Failure to have implemented restrictions early in the pandemic, and lifting of these PH measures too early (before a sufficient proportion of the population became immune due to vaccinations), may have resulted in catastrophic outcomes in terms of deaths and an overwhelmed health system.

### Limitations

Limitations of this study include the likely under-ascertainment of cases, hospitalizations and deaths in surveillance data, and the use of a model that simulated the epidemic in an “average Canadian community” without accounting for regional variations in demography, contact rates and sensitivity to infection. However, the model outcomes appear conservative projecting circa 4.5 million cases for Canada as a whole in the “observed baseline” scenario (suggesting, with 3.3 million reported cases, an optimistic 73% ascertainment rate) but 18,000 deaths compared to the 38,000 observed. The model did not consider outbreaks with high transmission and high case fatality rates in health care and long-term care settings ((28)); therefore, infections, hospitalizations and deaths were underestimated in the counterfactual scenarios.

## Conclusion

Re-analysis of the COVID-19 pandemic and public health responses will be common in the coming months and years. While the response to COVID-19 in Canada may have been relatively effective, it was not perfect, and further studies, including more regional analyses for Canada, will be needed to learn from this pandemic. This will require examination of the broader impacts of COVID-19 (particularly Long COVID), the range of public health measures and unintended consequences of public health measures on health.

## Supplemental material

These documents can be accessed on the Supplemental material file.
